# A Real-Time Automated Defect Detection System for Ceramic Pieces Manufacturing Process Based on Computer Vision with Deep Learning

**DOI:** 10.3390/s24010232

**Published:** 2023-12-31

**Authors:** Esteban Cumbajin, Nuno Rodrigues, Paulo Costa, Rolando Miragaia, Luís Frazão, Nuno Costa, Antonio Fernández-Caballero, Jorge Carneiro, Leire H. Buruberri, António Pereira

**Affiliations:** 1 Computer Science and Communications Research Centre, School of Technology and Management, Polytechnic of Leiria, 2411-901 Leiria, Portugal; esteban.c.cumbajin@ipleiria.pt (E.C.); nunorod@ipleiria.pt (N.R.); paulo.costa@ipleiria.pt (P.C.); rolando.miragaia@ipleiria.pt (R.M.); luis.frazao@ipleiria.pt (L.F.); nuno.costa@ipleiria.pt (N.C.); 2 Instituto de Investigación en Informática de Albacete, 02071 Albacete, Spain; antonio.fdez@uclm.es; 3 Departamento de Sistemas Informáticos, Universidad de Castilla-La Mancha, 02071 Albacete, Spain; 4 Grestel-Produtos Cerâmicos S.A, Zona Industrial de Vagos-Lote 78, 3840-385 Vagos, Portugal; jorgecarneiro@grestel.pt (J.C.); leireburuberri@grestel.pt (L.H.B.); 5 INOV INESC Inovação, Institute of New Technologies, Leiria Office, 2411-901 Leiria, Portugal

**Keywords:** defect detection, deep learning, CNN, industrial surface, automatic surface inspection, quality inspection

## Abstract

Defect detection is a key element of quality control in today’s industries, and the process requires the incorporation of automated methods, including image sensors, to detect any potential defects that may occur during the manufacturing process. While there are various methods that can be used for inspecting surfaces, such as those of metal and building materials, there are only a limited number of techniques that are specifically designed to analyze specialized surfaces, such as ceramics, which can potentially reveal distinctive anomalies or characteristics that require a more precise and focused approach. This article describes a study and proposes an extended solution for defect detection on ceramic pieces within an industrial environment, utilizing a computer vision system with deep learning models. The solution includes an image acquisition process and a labeling platform to create training datasets, as well as an image preprocessing technique, to feed a machine learning algorithm based on convolutional neural networks (CNNs) capable of running in real time within a manufacturing environment. The developed solution was implemented and evaluated at a leading Portuguese company that specializes in the manufacturing of tableware and fine stoneware. The collaboration between the research team and the company resulted in the development of an automated and effective system for detecting defects in ceramic pieces, achieving an accuracy of 98.00% and an F1-Score of 97.29%.

## 1. Introduction

Defect detection or anomaly detection in industrial processes is an important procedure. Currently, manual and visual inspections performed by experts are expensive due to the high cost of human labor during working hours, the possibility of material waste, and the degraded quality of shipped products [1]. In contrast, the use of machine learning algorithms for automatic defect detection reduces labor consumption [2]. In recent years, automatic defect detection has played a critical role in the industry’s inspection process. It improves product quality and aids in maintaining control of the manufacturing process, such as approving or rejecting produced parts in factories. Additionally, it reduces material waste by including the rework and repair of parts [3]. According to the literature reviewed by Prakash et al. [4], CNNs demonstrate superiority in the quantity of existing articles, information extraction from images, and performance over traditional machine learning models such as Support Vector Machines (SVMs), Cellular Neural Networks, and various image processing algorithms. After conducting our systematic review [5], we found that CNNs are predominantly used to detect defects in metals but have also been shown to be effective on other materials such as wood, ceramics, and surfaces. Consequently, the use of CNNs for surface defect detection provides a stable foundation and serves as an ideal starting point for further research on new or less-studied surfaces, thereby opening up new research opportunities. Based on the available information, it is feasible to apply the same techniques, algorithms, and networks to novel surfaces. Our research focuses on ceramic surfaces, and we collaborated with an industrial partner to conduct experiments in a real manufacturing environment.

Our research addresses the issue of detecting defects in ceramic pieces and its implementation in an industrial environment, including all the challenges associated with the manufacturing process. Therefore, a comprehensive understanding of the ceramic manufacturing chain is crucial for enhancing quality control in the factory. In our case, we have established an extensive partnership with our industrial collaborator, which has provided us with valuable insights into their manufacturing procedures.

This study is part of an industrial quality control process that aims to detect anomalies and defects in pieces during manufacturing. The main objective of this process is to identify defective pieces for further evaluation. The process is centered on acquiring images and classifying them into categories, resulting in an image classification problem. Additionally, the process becomes challenging and more complex due to several factors, including the manufacturing of multiple types of ceramic pieces, the presence of various defects, a dusty environment, and the difficulty of detecting very small defects, even for trained workers.

Our team aims to establish a standard classification for common ceramic surface defects. We proposed, developed, and implemented an automated model for binary classification using a deep learning model with CNNs to detect defects in ceramic pieces. This system is capable of distinguishing between ceramic pieces with defects and ceramic pieces without defects by utilizing images captured by a computer vision system that was developed and implemented within the factory. These images are captured using an image acquisition module equipped with an industrial camera, a customized housing with dedicated lighting, and Raspberry Pi. The module was created and installed at our collaborator’s factory and is responsible for storing the images in a digital repository. We used the stored images to generate a high-quality, properly labeled dataset. These images were preprocessed, and the resulting dataset were appropriately balanced. Then, we created a CNN model for image classification using the pre-existing dataset. The model has the ability to make precise predictions with images captured by the industrial camera.

In summary, the overall contributions of this article are as follows:

- The development of an automated real-time defect detection system using machine learning and computer vision;
- To present a method for the preprocessing images, specifically those of ceramic pieces;
- The evaluation and selection of the most suitable CNN for defect detection in ceramic pieces;
- The primary difficulties associated with capturing images in a factory, including issues with lighting, focus, and image size, are detailed;
- Summary of the ceramic pieces manufacturing process, detailed in collaboration with our industrial partner and adaptable to a wide range of cases within this sector.

This paper is divided into several sections. Section 2 presents the related work. Section 3 provides a summary of the manufacturing process. In Section 4, we provide an overview of the system, along with basic concepts and details of our methodology for addressing the problem. Next, Section 5 details the experiments and results, while Section 6 provides a discussion. Finally, we draw our conclusions and outline future work in Section 7.

## 2. Related Work

Studies were selected based on predefined criteria, including relevance to the topic, methodological rigor, and contribution to the advancement of knowledge in the field of surface defect detection. A systematic review process was used to carefully examine the objectives, methodology, results, and conclusions of each selected study. This approach facilitated a thorough and comparative assessment of the studies, culminating in our systematic review [5], which provides significant insight into current advances in surface defect detection.

Identifying defects in the manufacturing process is critical for companies because it has a direct impact on the quality and functionality of products. This makes defect inspection an integral part of the manufacturing process [6]. The most frequent defects found in most publicly available datasets are roll scratches, holes, cracks, patches, pitted surfaces, inclusions, and rolled-in scales. These defects are mainly found on metal surfaces and can serve as a guide for further studies on materials or surfaces. The five most prevalent types of surfaces, categorized from our systematic analysis [5], are metal, building, wood, ceramic, and special surfaces. The industry has conducted the most studies on metal surfaces.

The adoption of customized networks has gained popularity, with notable examples including the method proposed by Zhou et al. [7]. In their work, the authors advocate for the use of a streamlined CNN based on AlexNet and SDD (surface defect detection) for quality control. Another example is the CNN introduced by Gai et al. [8], which leverages VGG-16 for detecting surface defects in industrial steel parts. Defect detection in building surfaces is crucial to preventing structural failures. These defects can indicate aging, deterioration, or internal structural flaws [9]. The methods used to collect images for the datasets are noteworthy because of the challenges faced during acquisition. For instance, obtaining images from elevated locations such as bridge piers, tall buildings, and high-rise concrete structures requires specialized equipment. Saeed [10] used a quadcopter-type UAV equipped with a GPS and a camera for this purpose. Although wood is one of the most commonly used materials in industry, it remains understudied. Among the discovered studies are Ding et al.’s [11] proposed technique, which employs industrial cameras and supervised lighting to capture images, and Jung et al.’s [12] technique for generating artificial datasets. Some surfaces have not been well-studied due to their infrequent use in the industry, but they have been effectively incorporated into methods used for more commonly studied surfaces. For instance, Zou et al. [13] presented a study on defect detection on the painted surfaces of ancient Chinese buildings, while F. Xu et al. [14] developed a method for defect detection in paint film for anticorrosion and the decoration of metal workpieces. Ceramic surfaces detect defects such as cracks, bubbles, scratches, and burrs to reduce quality failures in industrial processes. To improve inspection and reduce material waste, automated methods have recently been adapted [1,15]. Our study focuses on ceramic surfaces, and we are guided by methods such as the one proposed by Min et al. [15]. This method aims to classify defects, including cracks, burrs, and bubbles based on their size, using CNNs and a dataset obtained through data augmentation techniques. Additionally, we consider the method introduced by Birlutiu et al. [1], which relies on image preprocessing and a custom CNN to predict images with and without defects.

This paper proposes a new approach for defect detection in ceramic pieces, with a machine learning model based on the information collected on the different types of surfaces found in our systematic review, performance improvement techniques, and image preprocessing. Research on ceramic pieces is scarce, focusing mainly on network comparisons or postmanufacturing analysis. We did not find a specific real-time defect detection system designed specifically for an industrial environment at this stage of manufacturing. However, similar studies on other surfaces have guided our system, adapted to the specific constraints of ceramic pieces (lighting, camera specifications, image dimensions, and different techniques). We stand out by offering a detailed and reproducible system, which is valuable given the unique nature of this material compared with more studied surfaces. Nonetheless, the confidentiality of the dataset is maintained. This is due to agreements with our industrial partner. We provide a comparison of three techniques (training from scratch, transfer learning, and transfer learning followed by fine-tuning), selecting the one with the most effective results. Next, we apply the selected technique to three different networks (AlexNet, VGG, and ResNet), comparing their outcomes with the objective of identifying the best-performing real-time model. For the ceramic piece images, we suggest a particular preprocessing method before using them in training the CNN. Results from experiments indicate that the chosen model’s performance and image preprocessing are reliable and perform well for detecting defects in ceramic materials.

## 3. Ceramic Manufacturing Chain

**Figure 1:**
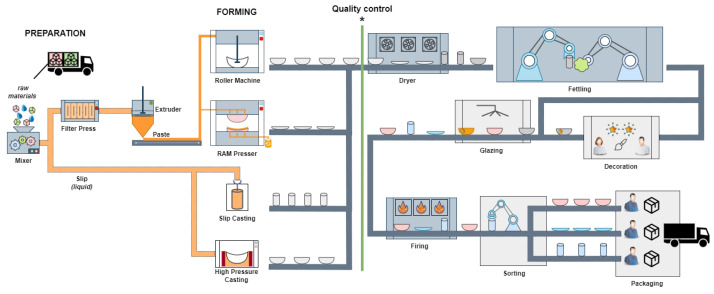
Ceramic pieces manufacturing process with quality control stage localization (*).

Stoneware is a ceramic material characterized by its durability, strength, and diversity of applications. It consists of a homogeneous mixture of clays, feldspar, and silica. The manufacturing process of stoneware involves several steps, from preparing the raw materials to the final firing of the ceramic, as shown in Figure 1.

After the preparation stage, the forming stage begins, in which molds, pastes, and calibrators are used. The choice of mold material, whether plaster or polymer, depends on the specific forming technology employed. This industrial process employs four forming technologies: roller, RAM (Rapid Adaptive Manufacturing) pressing, slip casting, and high-pressure casting. Quality control is implemented manually through human vision inspection before the drying phase. The labor-based human quality control process will be replaced by the automatic computer vision quality control system. This quality control is marked with an asterisk (*) in Figure 1. The next stage is decoration, which involves applying paints and employing various techniques to create desired effects on the ceramic pieces. Following decoration, the glazing stage is divided into two substages. More complex ceramic pieces are dip-glazed, where an operator immerses the ceramic pieces in the glaze. On the other hand, simpler ceramic pieces can be spray glazed manually using machines or robots. Finally, the ceramics are fired at temperatures ranging from 1150 ${}^{\circ }$C to 1200 ${}^{\circ }$C to prepare them for storage and distribution. Figure 1 illustrates the complete manufacturing process.

### 3.1. Forming

Ceramic tableware is produced by different forming methodologies described in Figure 1. The RAM press is advantageous for preparing small series due to cost-effective mold development and shorter manufacturing times. However, it generates a significant amount of waste due to mold overflow. Depending on the type of tableware, jiggering is used for round pieces such as mugs and plates, while ram pressing is suitable for various geometric forms like squares, triangles, and rounds. Slip casting in plaster molds enables the manufacturing of complex forms and hollowware. However, it is time-consuming and generates waste, similar to RAM pressing. In contrast, HPC (High-Pressure Casting) produces high-quality pieces while minimizing waste and achieving the same level of complexity as slip casting, except for hollowware. The exceptional quality of HPC is due to the use of polymeric-based molds, resulting in smoother surfaces and fewer surface defects caused by mold irregularities. Compared with slip casting, HPC offers faster manufacturing cycles. Jigging and jolly (roller) equipment is used to produce round parts. Each of the four forming technologies listed in this study requires a specific type of mold. Slip casting uses plaster molds, which have the greatest porosity. HPC uses polymer molds. The RAM press and roller techniques both require plaster molds, with roller molds providing the greatest mechanical strength. After the forming process, the ceramic pieces undergo a two-stage fettling process. The initial step is deburring, which entails the elimination of excess paste in the region where the molds were joined. The subsequent stage is sponging, which utilizes a moist sponge to remove any imperfections. The previously described quality issues with the demolding must be dealt with in this step. The variety of pieces coming out of the drier at the same time, requires equipment able to identify and fettling pieces according to the established protocols. Currently, this quality control process is carried out manually; therefore, our proposal is made at this stage of the manufacturing process.

### 3.2. Decoration

This stage of manufacturing involves the application of paints, engobes, granules, and other materials through manual techniques such as carving, sponging, troweling, and cutting to create decorative effects on pieces. It is one of the most complex stages of the manufacturing process, as these effects can be applied before glazing, between two different glazes, and/or after glazing.

### 3.3. Glazing

Glazing is the process of applying a layer of glaze to ceramic pieces. There are two methods of glazing mentioned below.

Dip glazing is used for more complex pieces (for example hollow or very closed parts) where the spray does not reach the interior (e.g., mugs, teapots, and pitchers). The equipment secures the piece via suction cup under vacuum and submerges it into glaze while rotating to achieve uniform coverage. The dip time and rotation speed depend on the type of piece being glazed. After removal, the operator places the ceramic piece in a small fountain to glaze the bottom. Subsequently, the operator passes the piece over a rotating wet sponge mat, removing the glaze that remains on the bottom of the piece, which must always be free of glaze. Spray glazing can be performed manually (in specific situations), applied in circular machines, or applied by a robot. In all three approaches, the pieces are placed on rotating supports, and manual glazing is performed manually by an operator. In the case of circular equipment, the rotation is automatic with stationary spray guns (manually tuned by an operator). In the last case, the glazing is performed by a robot applying the glaze in a predetermined way. The glaze suspension is applied using compressed air guns and is circulated through pumps that maintain the glaze in agitation.

### 3.4. Firing

The glazed and decorated pieces are placed on trolleys and manually loaded onto refractory slabs that are attached to wagons. The majority of the manufacturing is fired using continuous kilns that are fueled by natural gas. The pieces are fired at temperatures between 1150–1200 ${}^{\circ }$C.

## 4. Materials and Methods

The implementation of this system, together with all the materials, was carried out in the facilities of Grestel S.A., a factory specialized in the production of ceramic pieces for the international market. This company is located in the industrial area of Vagos, Portugal.

This article presents a methodology for identifying flaws or defects in ceramic pieces in real time within an industrial environment, reducing losses and improving product quality. The methodology involves implementing a solution for acquiring and storing images during the manufacturing process. A user-friendly labeling process is employed to construct an image dataset for the training stage of the CNNs. The following subsections present an exhaustive account of the methodology applied in this study. The section begins with a system overview, followed by an explanation of the image acquisition process and dataset creation. Following this, we examine the CNNs used in this study and conclude with a detailed description of the training methods employed.

### 4.1. System Overview

This paper presents a methodology aimed at real-time defect detection in ceramic pieces, with the overarching goals of minimizing losses and enhancing product quality. The methodology employs convolutional neural networks (CNNs) and specialized image preprocessing techniques designed specifically for ceramic pieces. Its applicability is particularly relevant for factories seeking to automate their quality control processes, given the similarities observed during the forming stage in most cases. The methodology was successfully implemented at our partner’s industrial facilities.

**Figure 2:**
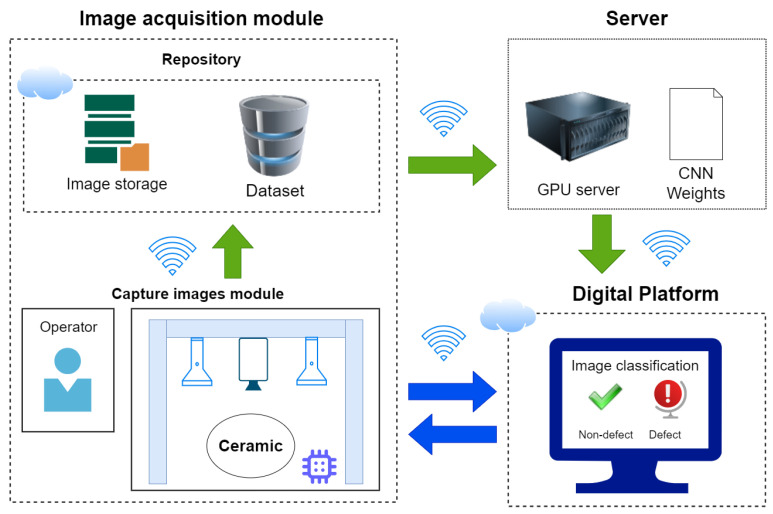
General solution architecture for model training and deployment.

The system comprises several crucial components that collaborate to achieve the desired outcomes. Firstly, a digital platform is integrated to enable real-time visualization of the defect detection process. This platform provides an interactive interface for monitoring the ongoing detection activities. Furthermore, a central repository is deployed to store all captured images during the defect detection process. This resource is valuable for data management and analysis. For accurate labeling of the images, an easy-to-use dashboard is implemented. This dashboard enables users to assign accurate labels to images, facilitating the training and validation phases of the CNN model. In addition, the image acquisition module is seamlessly integrated into the factory infrastructure and captures high-quality images of ceramic pieces, which serve as input data for the defect detection system. The proposed system combines essential components to present an effective and complete solution for real-time defect detection in ceramic pieces. The result is reduced losses and improved product quality. The methodology, as shown in Figure 2, comprises two distinct phases. The initial phase, which includes the flow of the green arrow, involves creating and training a CNN model that the platform uses to generate predictions. This phase commences with the image acquisition module, which captures images that are later stored in a repository to form a labeled dataset. The final CNN model is trained on a GPU server.

Once the model is created, the second phase follows the bidirectional flow of the blue arrow between two components and includes using it to detect defects. The camera in the factory captures an image of the forming process, which the system receives through the image acquisition module. The received image is preprocessed to extract meaningful information. The system then provides a prediction for the ceramic piece based on the processed image. This prediction provides crucial feedback in the form of an alert to the operators, indicating the presence of defects on the surface of the ceramic piece. In response to the alert, operators can swiftly discard the identified defective ceramic piece. Thus, the proposed system helps to reduce manufacturing costs and improve the overall quality of the product.

### 4.2. Defect Types

**Figure 3:**
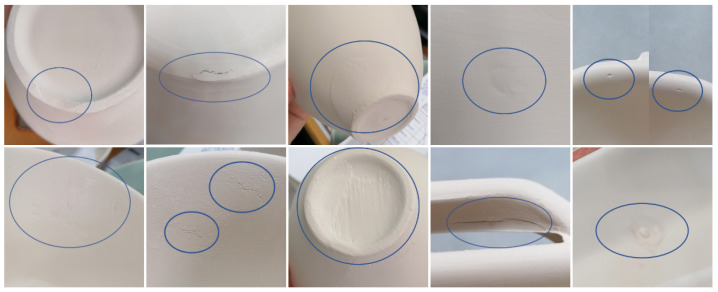
Examples of different defect categories that arise during the manufacturing process.

During the ceramic forming stage, factory personnel analyze the surfaces of the pieces to define the most frequent defect types, along with one type for the pieces without defects. These defects are marked with blue circles in each quadrant of the image, as shown in Figure 3. The challenge lies in acquiring a substantial number of images for classification. Certain defect types occur more frequently than others, leading to the conclusion that the entire range of defects in the dataset will fall under the “defect” category. The “nodefect” category refers to ceramic pieces that are not defective. The captured image will always show a complete ceramic piece for a comprehensive inspection.

### 4.3. Image Acquisition

**Figure 4:**
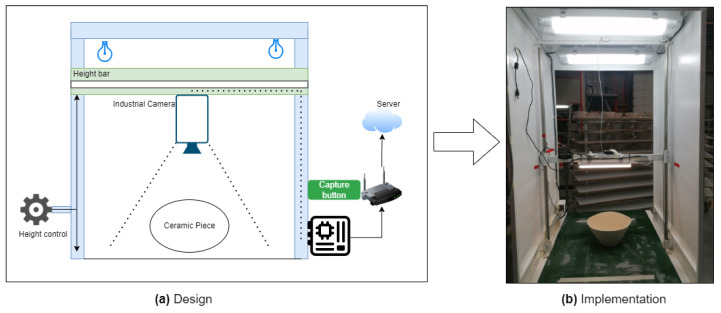
Image acquisition module: schematic representation and real image of the manufacturing scene. Image acquisition system design (**a**). Image Acquisition system installed at the factory (**b**).

The camera is the most essential element of the image acquisition module. Selecting the appropriate camera enhances dataset quality, thus increasing defect detection accuracy. We chose an industrial camera due to its superior quality compared with webcams and conventional cameras. It offers a frame resolution of 4024 × 3036. The camera is equipped with dedicated software that optimizes image capture through customizable parameters. Users can select lenses according to their needs to ensure greater focus and image quality. In the present scenario, we used a MER-1220-32U3C camera with a 6 mm focal length. The physical image capture module design is shown in Figure 4a, with Raspberry Pi at the center of the connections. It receives images from the industrial camera and sends them to the digital repository located on our server. The camera was positioned on the height bar, allowing for easy selection of the ideal distance for acquisition based on the type of ceramic pieces. This approach eliminates the need for zoom, which we avoid due to its potential interference with real-time quality.

The camera lens settings are manually set and remain fixed to ensure that the dataset images have the same characteristics as those used for predictions. The physical infrastructure of the image acquisition module is shown in Figure 4b, highlighting the manually configured stable illumination. The module connects to the image repository via the internet and Raspberry Pi 4. The source code is hosted on Raspberry Pi 4 developed using the official documentation of the industrial camera. This module is used to generate an image dataset and capture images in real time during the manufacturing process to detect defects.

**Figure 5:**
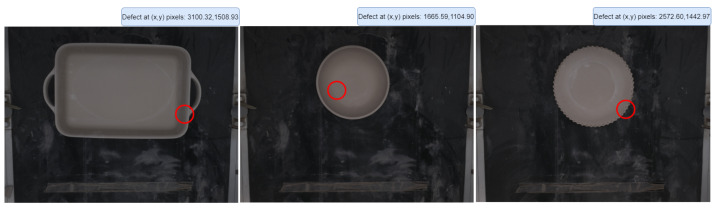
Developed Dashboard for image labeling.

The images for the datasets are stored in a platform named “Dashboard” developed by the team. This platform stores and labels images in a repository so that they can be processed to create the dataset used for CNN training. An illustration of the Dashboard labeling by the factory personnel is shown in Figure 5. This method stores the defect’s coordinates in each captured image. A red circle is drawn using the coordinates of the defects that we had captured as the center, thus showing where the defect is located. This approach allows us to easily identify any defects that are not initially visible. It can also aid in labeling if we need to use an object detection algorithm.

**Figure 6:**
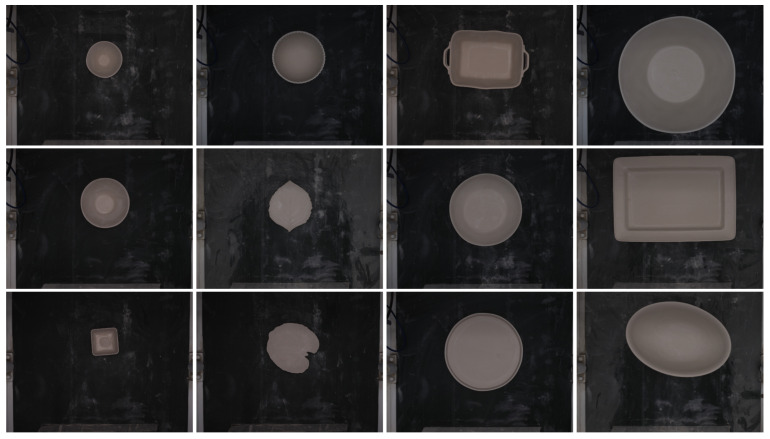
Samples of ceramic pieces with different sizes stored in the repository.

Finally, Figure 6 displays several examples of the images captured and stored in the repository, showcasing the wide range of ceramic pieces produced by the factory. In this case, we focus on the top 10 most commonly produced ceramic pieces within the factory. Due to their differing sizes, the varied ceramic pieces pose a challenge during CNN training as their defects are less apparent. Therefore, it is imperative to preprocess the images before developing the dataset.

### 4.4. Image Preprocessing

Image preprocessing for CNNs in the context of classification problems involves the application of a variety of techniques to images prior to their input into the CNN model. The aim of image preprocessing is to improve image quality, with the ultimate goal of improving CNN performance in classification tasks. In this particular case, we convert the initial RGB image obtained from the industrial camera into an image with uniform dimensions. It is critical for CNN models to have a fixed image size, which ensures uniformity across all input images. Additionally, we maximize the space of the ceramic piece within the image.

**Figure 7:**
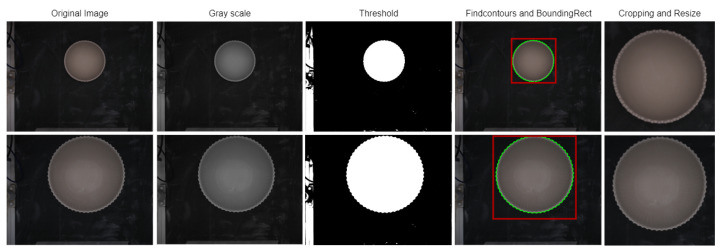
Image preprocessing pipeline from the original image to the cropped and resized image.

Our four-step preprocessing process, depicted in Figure 7, involves RGB to grayscale converting, thresholding, contour detection, and cropping and resizing of the original RGB image. Initially, the image representation is transformed from the RGB color space to the grayscale space, which is represented by luminance (*Y*), as shown in Equation (1). The coefficients 0.299, 0.587, and 0.114 are commonly used to convert RGB to grayscale, as recommended by ITU-R BT.601-7. These coefficients reflect the relative sensitivity of the human eye to each of the primary colors. Notably, these formulas match the functions of PyTorch, where our experiments were performed.

(1) RGB[A]toGray:Y←0.299·R+0.587·G+0.114·B

The grayscale image is then used to apply the *THRESH BINARY* operation based on the Equation (2). Where, src(x,y) represents the current pixel value, and T(x,y) represents the threshold value for each pixel. Furthermore, maxValue is assigned to pixel values in excess of the threshold. Careful illumination control of the image acquisition module ensures that T(x,y) remains consistent across ceramic pieces, regardless of their shape. This consistency is due to the uniformity of both the pieces’ material and color.

(2) $$\mathrm{dst}\left(x,y\right)=\left\{\begin{array}{cc} \mathrm{maxValue}, & \mathrm{if}\;\mathrm{src}(x,y)>T(x,y)\\ 0, & \mathrm{otherwise} \end{array}\right.$$

The subsequent step involves obtaining the contours, which is achieved by using the contour detection algorithm called *findContours*, developed by Suzuki and Be [16] within OpenCV. This algorithm generates an array of contours for the objects present in the image. The largest contour, which corresponds to the ceramic piece in our specific case, is indicated by the green line. The we use OpenCV’s *boundingRect* function to extract the coordinates of a bounding rectangle. The ceramic piece is bounded by a red highlighted rectangle.

As a result, we achieved our objective of obtaining the four coordinates needed to crop the original image. The resulting image includes the ceramic piece and a small margin that will later be resized to a fixed size. Afterwards, the entire dataset must be resized, taking into consideration the size of the smallest image, to guarantee that all images have the same dimensions. This process is highly dependent on the camera used and the size of the ceramic piece. A positive aspect of this approach is that it yields a dataset that is compatible with different networks.

### 4.5. Data Augmentation

**Figure 8:**
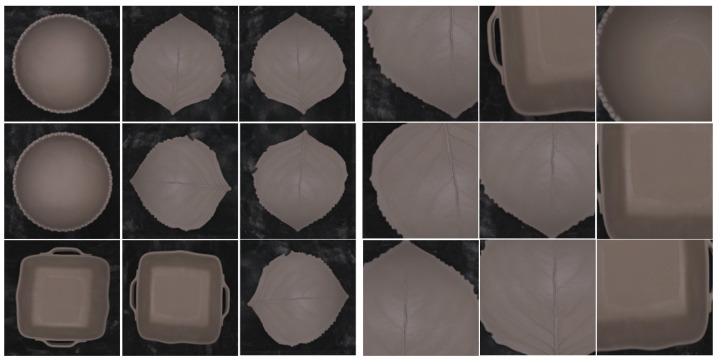
Correct (**left**) (using flip and rotation) and incorrect (**right**) data augmentation using Zoom.

Data augmentation is an important technique when working with small datasets to enhance outcomes [17]. It helps prevent overfitting and underfitting problems during training by expanding the range of datasets and helping the model identify important patterns. By augmenting the available data, the model can enhance its robustness, adaptability, and ability to achieve superior generalization performance [18]. This methodology facilitates the artificial generation of new images in an efficient and convenient manner, using techniques such as perspective skewing, elastic distortions, rotation, shearing, cropping, and mirroring [17]. However, for our specific case, we decided to limit the augmentation process to rotation and flipping. This decision is motivated by the observation that important details can be lost then zooming, especially at the periphery of ceramic pieces. This poses a challenge because our datasets must contain defects in these edge regions. As shown in Figure 8, implementing a zooming technique results in missing defects, leading to imprecise training data and potential misclassification. Therefore, we prioritize the chosen augmentation methods to preserve critical defect information during the training process. This is executed prior to the training stage by generating a fixed number of images for each original image, which distinguishes it from other data augmentation techniques that apply random transformations at the batch level during the training stage.

### 4.6. Transforms and Normalization

Transforms and normalizations are often used at the batch level during the training phase to enhance model generalization and convergence [19]. Transforms are used as data augmentation techniques to randomly alter images within a batch before inputting them into the model for training. In our system, we implement random small rotations within the range of [−15; +15] degrees and flips as data transformations. By utilizing batch data augmentation, we increase the diversity of the training data within each batch and epoch, ultimately reducing overfitting and enhancing the model’s capacity to generalize to unseen data. The second method, referred to as “normalization” by Finlayson et al. [20], involves removing dependencies caused by lighting geometry and illuminant color. Deininger et al. [21] describe normalization as a process that expands or reduces the range of pixel intensity values by manipulating either the intensity level or the entire range. The goal is to ensure uniform intensity levels among all images. To achieve this, we apply the mean and standard deviation within the normalization function after scaling pixel values to a range of [0, 1]. As per [22], the normalization of image datasets enhances network training by reducing internal covariate shift.

Standardizing all three color channels (RGB) requires calculating the mean ($\bar{u}$) by summing the pixel values of each image in the dataset and dividing by the number of images (*N*) using Equation (3). The previously calculated mean is then used to determine the standard deviation (σ) in Equation (4).

(3) $$\bar{u}=\frac{1}{N}\sum_{i=1}^{N}u_{i}$$

(4) $$\sigma =\sqrt{\frac{\sum_{i=1}^{N}{\left(u_{i}-\bar{u}\right)}^{2}}{N}}$$

Mean and standard deviation are calculated for each individual dataset. This is necessary because the lighting conditions varied throughout the project. Equation (5) is used to normalize each pixel (*x*) of the image.

(5) $$x:=\frac{\left(x-\bar{u}\right)}{\sigma }$$

**Figure 9:**
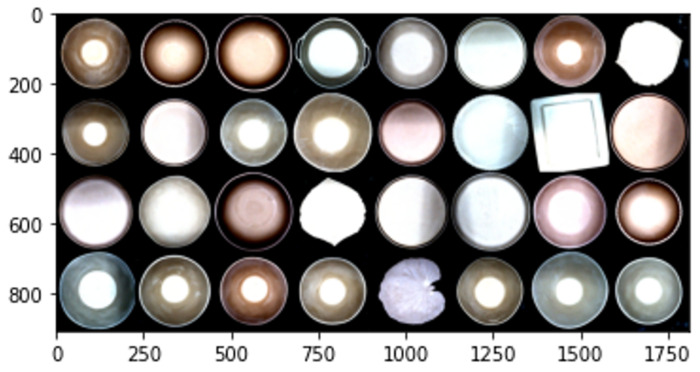
Samples of random transforms and normalization during the training.

The images shown in Figure 9 are the output of the normalization and transform application. These images are a product of the random flips and rotations that were applied, followed by normalization.

### 4.7. Networks Architecture

**Figure 10:**
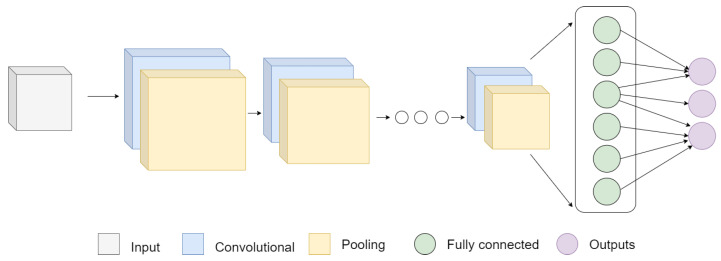
General structure of a CNN with convolution layers, normalization layers, pooling layers, and fully connected layers.

The CNNs used in this study follow the foundational architecture illustrated in Figure 10. These networks leverage different types of layers such as convolution, normalization, pooling, and fully connected layers to enable deep learning, resulting in remarkable outcomes for defect detection. For our experiment, we chose three well-known networks and implemented three specific techniques, which are elaborated in subsequent subsections. Our objective is to determine the top-performing network for integration into our final model.

We chose three of the most extensively used neural networks based on our systematic review [5] and the recommendations provided by Kermanidis et al. [23]. Although there is a lack of studies focused on ceramic pieces, it is worth noting that ResNet [15] and VGG [24] have been successfully implemented by researchers. Additionally, we included AlexNet in our selection due to its abundance of information and studies showcasing successful defect detection. The detailed descriptions of the selected networks are provided below.

First, AlexNet was developed by Alex Krizhevsky in collaboration with Ilya Sutskever and Geoffrey Hinton, specifically for the ImageNet Large-Scale Visual Recognition Challenge (ILSVRC-2012) [25]. Technical abbreviations will be explained upon their first usage. This convolutional neural network (CNN) consists of five convolutional layers with max pooling operations and is followed by three fully connected (FC) layers [26]. Second, there is the Visual Geometry Group (VGG), which was proposed by Simonyan and Zisserman [27] from the University of Oxford for the ImageNet Challenge 2014. Dhillon and Verma [28] mention that VGG stands out for being a simple and deep network because it uses very small convolution filters (3 × 3), and every hidden layer has a rectification nonlinearity function, so it obtains good results on the classification of images and their localizations. Finally, the deep residual network (ResNet), created by He et al. [29] in 2015 and winner of the ILSVRC 2015 classification task, has gained recognition for its groundbreaking performance in training hundreds or thousands of layers while retaining excellent results.

### 4.8. Training Methods

Different training techniques aim to address common challenges in neural network training, such as early overfitting and lack of training images. Two main concepts are used to overcome these problems. The first technique is called “freezing layers”, which preserves the weights of specific chosen layers, frequently the initial layers of a pretrained model, to avoid alteration. On the other hand, the concept of “unfreezing layers” enables modifications and retraining of layers in a pretrained model [30]. Our experimental process uses three commonly employed approaches to train neural networks: training from scratch (TFS), transfer learning (TL), and fine-tuning (FT).

#### 4.8.1. Train from Scratch (TFS)

**Figure 11:**
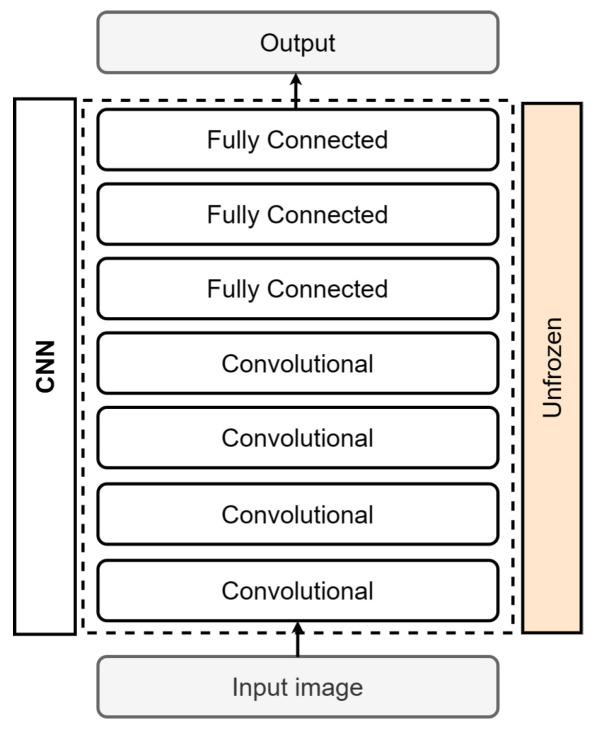
CNN from scratch.

When starting from scratch, a neural network is first randomly initialized and then trained on your specific task and dataset, as depicted in Figure 11. The network learns task-specific features and parameters from scratch, without relying on pre-existing knowledge or models. Another approach is to use a network architecture without transfer learning and use a random weight initialization, as described by Boyd et al. [31]. This method consumes more computational resources, time, and new data than using a network with transfer learning or fine-tuning, which uses weights created in pretrained models [32]. According to Ali et al. [9], establishing a reliable CNN from scratch entails extensive training with a sizable and resilient dataset. Although widely used techniques such as transfer learning and fine-tuning exist, some studies demonstrate that training from scratch produces better results than using a pretrained model. Shi et al. [33] and Bethge et al. [34] provide evidence to support this claim. This training method is used to address a problem with data that are not within the knowledge of a pretrained model.

#### 4.8.2. Transfer Learning (TL)

**Figure 12:**
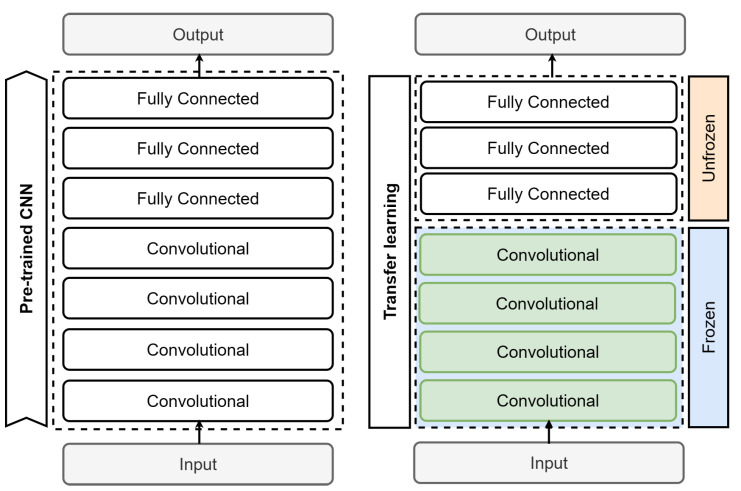
CNN training with transfer learning.

Transfer learning is a widely used technique in machine learning that is typically employed when working with small datasets, limited time, or high computational processing costs, as datasets are often difficult to create and require significant financial investment. This methodology is based on using previous knowledge gained from solving similar problems and applying it to a new problem with comparable characteristics [32]. Rather than beginning from a random starting point, we employ the weights from the pretrained model to initiate the process. The notion is that the pretrained model has already understood general functions that are transferable to the task at hand. The initial step usually entails replacing the output layer with one tailored to a specific problem. Next, the training process involves unfreezing the last layer, or a portion of the network, usually the classifier comprising the top three fully connected layers, as depicted in Figure 12.

#### 4.8.3. Transfer Learning with Fine-Tuning (FT)

**Figure 13:**
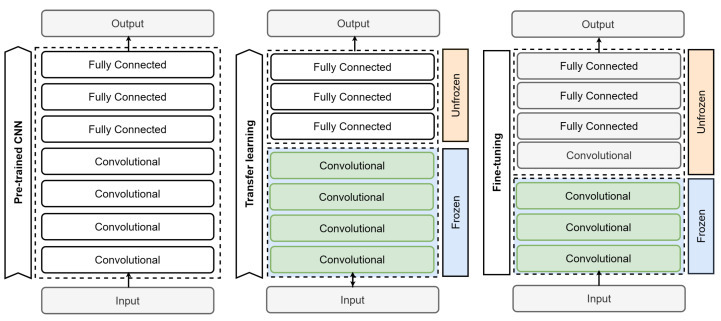
Training CNN model using fine-tuning.

Fine-tuning is a technique that follows transfer learning. It entails adjusting the weights of a pretrained model on a specific dataset for a given task. Instead of training the entire network from scratch, certain layers of the pretrained model are selectively unfrozen and updated while the others remain frozen. This allows the model to adapt its learned representations for the new task while maintaining the general knowledge acquired during the pretraining phase. Fine-tuning can be beneficial when working with smaller datasets or when the pretrained model’s knowledge is highly relevant to the task at hand [35,36]. The process of fine-tuning involves adding our custom network on top of an already pretrained base network, extracting features, freezing it, and training the remaining layers, typically the classifier. Then, the created model must be trained again. So, some layers in the base network are progressively unfrozen and trained together with the classifier. This process is illustrated in Figure 13. First, we initialize a pretrained CNN and apply transfer learning to train the classifier layers. The trained model is then saved for future use. When training with new adjustments, the objective is to enhance the process by incorporating the cumulative knowledge of the model. Therefore, the model is retrained by unfreezing an additional layer, specifically the last convolutional layer of the feature extractor.

## 5. Experiment and Results

Several experiments were conducted using Python to assess the performance of the proposed system and to identify the most effective combination of training techniques and CNN network architecture for detecting defects in ceramic pieces on a real manufacturing line. Three deep learning training techniques outlined in Section 4.8 were compared, and the most appropriate one was used to train, evaluate, and compare three of the most well-known CNN architectures. To ensure a robust working system, it is important to conduct a concise training process. Comparable training accuracy and validation are desirable, and the training loss should be similar to or slightly lower than the validation loss [9].

### 5.1. Dataset

**Figure 14:**
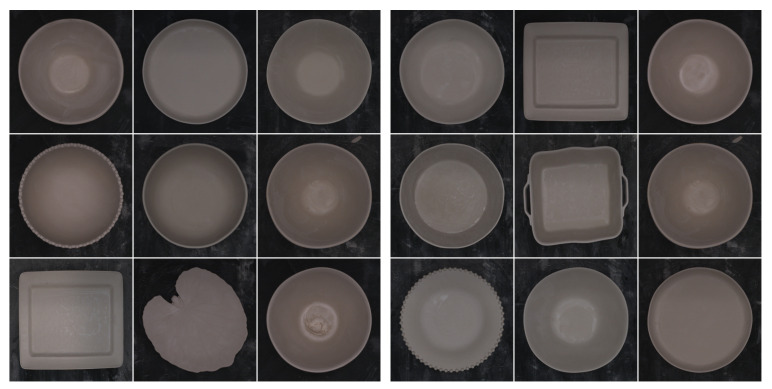
Samples of defect class (**left**) and samples of nodefect class (**right**).

The dataset used in the study includes images taken at the manufacturing line (as shown in Figure 14), using the labeling tool we developed. To streamline the images for both training and testing, we concentrated on the ten types of continuous manufacturing in the factory and subsequently categorized them into the two classes mentioned above.

The dataset was divided into training, validation, and test subsets. The training subset consisted of approximately 80% of the data, while the validation and testing subsets each consisted of 10%. Given the challenges encountered during image acquisition, we strove for balance and accomplished this by incorporating 374 images for the “defect” category and 294 images for the “nodefect” category within the training subset, while the testing and validation subsets each comprised 50 images per class. To balance and enhance the training set, we utilized data augmentation techniques to generate 2000 images for each class. Figure 14 shows an example of the dataset where each image solely depicts the ceramic piece with a slight border.

### 5.2. Techniques Comparison

The primary aim of the initial set of experiments is to determine the optimal training technique and to gather quantitative data on performance outcomes. To study the training methodology, we used the AlexNet architecture in our defect detection system. The selection of this architecture is based on its recognized reputation, straightforward analysis process, availability of ample documented information, and multiple successful use cases found in the image classification literature. Previous studies, such as the one in [37,38,39], have demonstrated the efficacy of this network in tackling similar problems, further supporting our choice.

The results presented in Table 1 were obtained with 250 training epochs, except for the last row, which pertains to the FT technique, where only 200 epochs were used. TL unfreezes three dense layers of the classifier and achieves remarkable results, despite the ceramic pieces having distinct characteristics from the images that generated the pretrained model. Next, FT uses the weights generated by TL for retraining. However, a significant difference in this scenario is that the final four layers are unfrozen, resulting in additional enhancements. As a result, there is a steady pattern of enhancement as the networks are modified.

**Figure 15:**
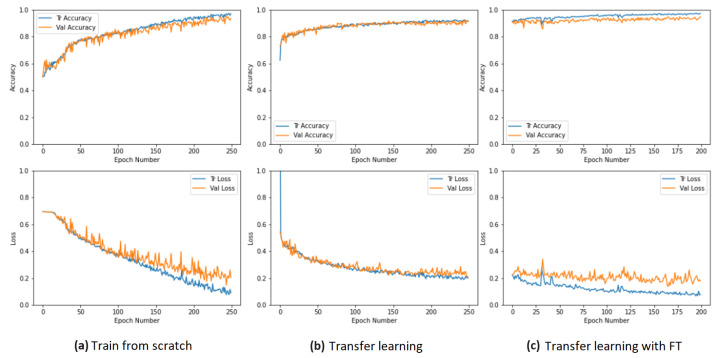
Accuracy and loss curves during the training process for the three techniques used.

The training processes and the corresponding accuracy and loss curves for the training and validation sets are presented in various charts in Figure 15. The accuracy curve for TFS shows instability at the beginning, while the loss curve exhibits variations in the validation values. Nevertheless, both curves appear stable, as depicted in Figure 15a. In the TL method, the accuracy curve for both training and validation sets continued to improve, as seen in Figure 15b. Throughout the FT process, the accuracy curves for training and validation maintain an upward trajectory, starting from the final values of the transfer learning and reaching the highest values among the three training methods, as shown in Figure 15c. We also analyzed the loss curves for FT with 250, 300, and 400 epochs and found that the loss curve for 200 epochs is the most stable with the least overfitting.

After training, the best models were periodically evaluated using the validation set and stored for carrying out quantitative testing using the F1-score metric (6).

(6) $$F1-\mathrm{score}=\frac{2\times (\mathrm{Recall}\times \mathrm{Precision})}{\mathrm{Recall}+\mathrm{Precision}}$$

**Figure 16:**
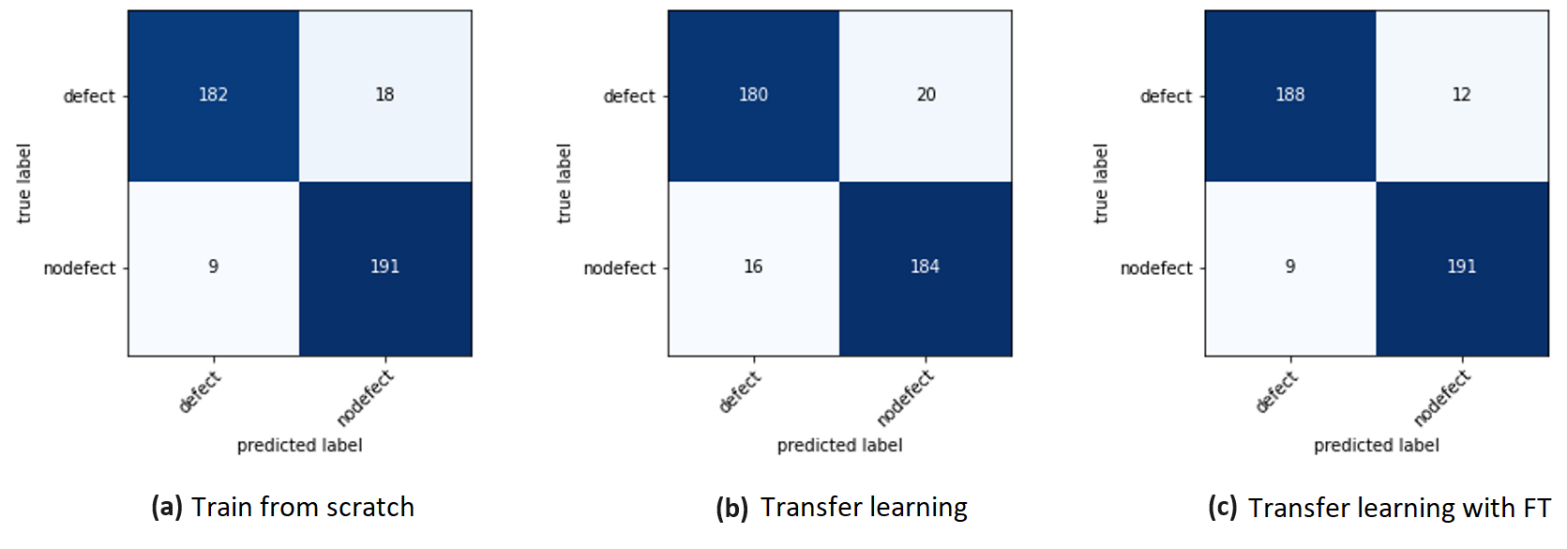
Confusion matrices.

These tests were conducted on images not previously seen by the models, which form the test set and are depicted in Figure 16. Confusion matrices were used for the analysis.

### 5.3. Network Comparison

The manufacturing line in this industry generates thousands of ceramic pieces per day. The aim of this study is to create a system that can instantly notify users to remove any substandard ceramic pieces from the manufacturing line. The subsequent tests seek to determine the ideal network, custom-made for image classification problems, that is most appropriate for our case study. The deciding factor in our selection is based on quantitative values that serve to validate our choice. In addition to the model used in the previous test set, our study incorporated two other convolutional architecture models, namely VGG11 [27] and Resnet18 [29]. These models are readily available in the official PyTorch documentation (https://pytorch.org/docs, accessed on 20 July 2023) and have demonstrated a high level of effectiveness and accuracy in a variety of image classification challenges within an industrial context, e.g., [40,41,42].

**Figure 17:**
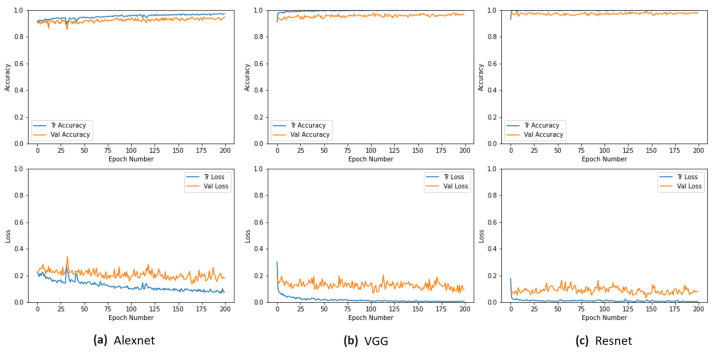
Accuracy and loss curves for 3 CNN models.

The three models were trained using the most effective methodology from the previous test set, which was FT. Figure 17 displays the accuracy and loss curves obtained during the training process. The results are comparable, and none of the three networks experienced overfitting issues.

The selected networks underwent the fine-tuning methodology with subtle differences that varied according to each network, following the identical steps adopted in AlexNet. Specifically, for VGG, the classifier and two convolutional layers were unfrozen, while for ResNet, the initial two sequential blocks needed to be unfrozen.

Table 2 presents the results that were obtained, wherein it was concluded that ResNet outperformed the others with a 98% accuracy rate and an F1-score of 97.2%.

**Figure 18:**
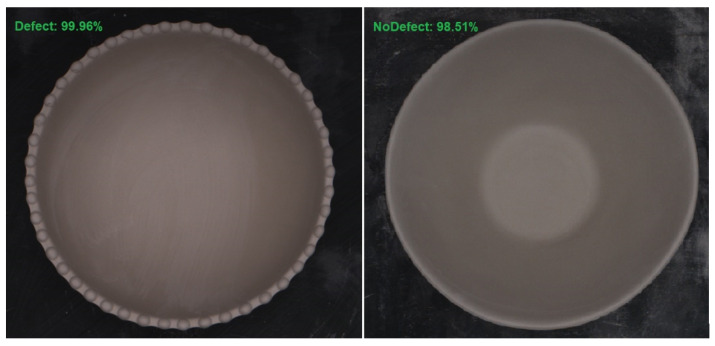
Prediction values for two sample images.

The system based on the ResNet architecture, trained with the FT method applied after the TL process, was tested under real conditions in a factory environment. In Figure 18, two examples of classification results generated by the neural network are shown, demonstrating a high level of confidence in the predictions made, i.e., above 98% for each image sample.

## 6. Discussion

The system resulting from the multiexperiment comparative study, which encompassed multiple experiments, includes an image preprocessing algorithm and a specific training model. The training model employs fine-tuning after transfer learning using the ResNet-adapted model. The automated defect detection system for ceramic pieces operates in real time and achieves impressive performance results. It has a testing accuracy of 98.00% and an F1-score of 97.29%, as evidenced in Table 2. The FT method enhances system performance, with the ResNet model demonstrating superior performance to other tested models.

The acquisition of a sufficient number of images to develop a comprehensive and trainable dataset is a vital aspect of systems and experiments in this field. Unfortunately, we experienced delays in this collection process. When applying this approach in an industrial environment, challenges arose during image acquisition due to the need to prioritize daily manufacturing demands and the limited time and resources available for experiments. As a result, we are currently limited to categorizing the available images into “defect” and “nodefect” groups. However, the increasing number of images presents an opportunity to improve our classification by generating new categories dependent on the types of ceramic pieces and defects.

The development of a suitable tool, demonstrated in Figure 5, for prompt and easy annotation significantly reduced the waiting time and facilitated the creation of a balanced dataset. In the initial stages of our experiments, we encountered the problem of overfitting caused by a lack of images. We addressed this problem by using data augmentation prior to training, followed by transformations and normalization during the training phase. The addition of more images led to a significant improvement that increased over time. Modifying the model to classify multiple categories of defects in ceramic pieces posed another challenge. To tackle this problem, we applied dropout to specific fully connected layers and employed input data normalization through mean and standard deviation. It remains experimental for future work due to the problem of insufficient images to expand to more than two classes but shows favorable results in binary classification.

One drawback is the requirement for high-quality images due to the small size and lack of contrast of defects in the ceramic pieces. Acquiring images in this type of system can be challenging in terms of lighting and calibration. Incorrect lighting control led to poor model performance in our early experiments, as the use of ambient lighting caused defects to be picked up by the camera depending on the time of day or environmental conditions, ultimately ruining many images. Thus, we found that static lighting in a controlled environment is the first step, as lighting variations cause noise and degrade image quality. Next, the camera should be placed at the ideal distance according to the manufacturer’s specifications. Lenses play an important role, and depending on the type of lens used, it is necessary to manually calibrate parameters such as aperture, focal length, minimum distance, and zoom, among others. We observed a difference in quality when using automatic white balance and static gain. Therefore, it is essential to have good knowledge of the subject and to experiment with the lighting to achieve a harmony of settings. Clear and detailed images yield better results.

Our industrial partner produces many types of ceramic pieces, including unique designs for custom orders and others intended for continuous production. Therefore, in this initial phase of our study, we focused on the 10 most common ceramic pieces, divided into two classes (defect and nodefect) to ensure a balanced dataset. This strategic selection minimizes the differences between parts by using common molds and suction cup types, achieving uniformity of defects and contributing to dataset standardization. We use all the generated images in the dataset, but it will be essential to balance the number of images for ceramic pieces with and without defects in future stages. A future automation of the image acquisition process will solve this time constraint associated with manual acquisition, which is currently limited to the available time of the company’s assigned personnel.

The fragility of the pieces is a consequence of the manufacturing process, in which the stoneware pieces are subjected to only one firing, in witch the ceramic body is fired in the kiln together with the glaze. Thus, the entire manufacturing process is carried out with fragile pieces and low mechanical strength. This requires the use of special equipment that only applies the force that is strictly necessary to handle the pieces through the various stages of forming, fettling, glazing, decoration, and firing. When analyzing the manufacturing process described in Section 3, one aspect stands out, namely the amount of manual work performed during this industrial process. With the exception of glazing, which is performed by a robot, all the operations shown in Figure 1 are carried out manually, from the handling of the pieces after forming to fettling dry pieces, glazing, decoration, loading the pieces into wagons that enter the kiln, unloading these wagons, sorting the final product, and packaging.

The defect detection system was integrated into the quality control framework, as shown in Figure 1. Previously, quality control was performed manually by an operator. Now, our automated system has replaced this process by issuing an alert when a defect is detected in a ceramic piece. After this detection, the operator performs a second validation and makes the decision to discard or keep the ceramic pieces. This approach facilitates human–machine collaboration, resulting in a significant improvement in the manufacturing process.

The manufacturing of tiles has a higher level of automation due to the tendency towards monomanufacturing, and the same is true for tableware when the manufacturing is more uniform. However, automating the stoneware tableware industry presents three main challenges: the complexity of products (including a variety of shapes, sizes, and decorations performed simultaneously), the fragility of the pieces, and the need for quality control.

Similar research on materials such as metal, concrete, wood, ceramics, and specialty surfaces exists and is detailed in our systematic review [5]. We identified common characteristics and challenges in these materials and applied them specifically to the ceramic context. Techniques such as data augmentation have proven valuable in improving our model’s performance. It is important to consider factors such as lighting issues for metallic surfaces, transfer learning for specialty surfaces due to image scarcity, and camera variations in concrete. This work contributes to improving our current model and lays the foundation for future research in this area. In Section 3, we explain the manufacturing process as a guide and source of information for small companies This information comes from a large company in the industry with large-scale manufacturing.

The results are consistent. Our main contribution is a new system that uses CNNs and industrial cameras, along with a web platform and a specific dataset we created. We also developed a method for image preprocessing of ceramic pieces that is capable of detecting defects in real-time industrial environments.

## 7. Conclusions and Future Work

The research team developed an automated defect detection system. The system includes an image acquisition module, a dataset generation for training purposes, and a convolutional neural network model. The dataset was generated using industrial cameras and carefully regulated lighting, and the system achieved an exceptional accuracy rate of 98.00% despite overcoming numerous challenges through extensive testing. Proper lighting is a crucial parameter to consider, as it directly affects the camera’s ability to capture defects in images. Regarding CNN training, the best results were achieved by using fine-tuning, data augmentation techniques, and calculating the mean and standard deviation for each dataset.

ResNet was selected as the convolutional neural network model due to its exceptional accuracy, achieving a testing accuracy of 98.00% and an F1-score of 97.29%. The F1-score metric is well suited to this problem domain as it effectively measures the network’s ability to detect defective ceramic pieces, also known as true positives (TP). ResNet surpassed the other two networks assessed and demonstrated the best results. Although ResNet is the deepest network of the three, VGG also demonstrated impressive performance for this particular surface-related task. The automation process is meaningless without an integrated system that allows for comprehensive management of the manufacturing process. Ensuring product quality control at various stages of the manufacturing line is a key factor in achieving this integrated control. The project specifically targeted quality control during the initial forming and fettling stages.

In the future, the lessons learned here will be applied to the other stages of the manufacturing process, specifically glazing, decoration, firing, and sorting. It is important to note that these stages will present much greater challenges than the one currently addressed. It is evident that the future of the ceramic industry will involve a more automated manufacturing process. The next step is to establish additional categories based on the specific types of ceramic pieces and other types of defects. This procedure entails identifying these types and then categorizing the defects inherent in each ceramic piece, automating the process, and facilitating the efficient organization and distribution of datasets. Unlike other industries, ceramics, with its distinctive organic shapes and inherent diversity, presents significant challenges that cannot be met by conventional methods used in more typical manufacturing lines. Tailored solutions are necessary to address the specificities of stoneware tableware. These solutions should not be prohibitively expensive to implement. Ceramic tablewares are not high-value products; therefore, any solutions developed must consider the cost and the ability of the solution to withstand industrial environments.

## Figures and Tables

**Table 1:** Training and test evaluation results for the 3 training methods.

Method	Epochs	Train Acc.	Train Loss	Test Acc.	Test Loss	Precision	Recall	F1-Score
TFS	250	96.84%	0.1107	93.50%	0.2142	95.28%	91.00%	93.09%
TL	250	92.53%	0.2114	92.25%	0.2201	91.83%	90.00%	90.90%
FT	200	97.28%	0.0934	94.75%	0.2172	95.43%	94.00%	94.71%

**Table 2:** Network models comparison using the FT method.

Method	Epochs	Train Acc.	Train Loss	Test Acc.	Test Loss	Precision	Recall	F1-Score
AlexNet	200	97.28%	0.0934	94.75%	0.2172	95.43%	94.00%	94.71%
VGG	200	99.58%	0.0137	96.33%	0.0936	95.42%	97.33%	96.37%
ResNet	200	99.83%	0.0041	98.00%	0.0791	98.63%	96.00%	97.29%

## Data Availability

In accordance with our research collaboration and data confidentiality agreement, the data used in this study are considered private and cannot be publicly shared. As such, we are unable to provide access to the datasets analyzed or generated during the research. We assure that the privacy and confidentiality of the data were strictly maintained throughout the study, adhering to ethical and legal considerations. While we are unable to make the data publicly available, we have followed the necessary protocols to ensure the integrity and validity of our findings.
